# Nanoparticle-Assisted Metabolomics

**DOI:** 10.3390/metabo8010021

**Published:** 2018-03-13

**Authors:** Bo Zhang, Mouzhe Xie, Lei Bruschweiler-Li, Rafael Brüschweiler

**Affiliations:** 1 Department of Chemistry and Biochemistry, The Ohio State University, Columbus, OH 43210, USA; zhang.5648@osu.edu (B.Z.); xie.392@osu.edu (M.X.); 2 Campus Chemical Instrument Center, The Ohio State University, Columbus, OH 43210, USA; bruschweiler-li.1@osu.edu; 3 Department of Biological Chemistry and Pharmacology, The Ohio State University, Columbus, OH 43210, USA

**Keywords:** nanoparticles, metabolomics, bioanalysis, NMR, MS

## Abstract

Understanding and harnessing the interactions between nanoparticles and biological molecules is at the forefront of applications of nanotechnology to modern biology. Metabolomics has emerged as a prominent player in systems biology as a complement to genomics, transcriptomics and proteomics. Its focus is the systematic study of metabolite identities and concentration changes in living systems. Despite significant progress over the recent past, important challenges in metabolomics remain, such as the deconvolution of the spectra of complex mixtures with strong overlaps, the sensitive detection of metabolites at low abundance, unambiguous identification of known metabolites, structure determination of unknown metabolites and standardized sample preparation for quantitative comparisons. Recent research has demonstrated that some of these challenges can be substantially alleviated with the help of nanoscience. Nanoparticles in particular have found applications in various areas of bioanalytical chemistry and metabolomics. Their chemical surface properties and increased surface-to-volume ratio endows them with a broad range of binding affinities to biomacromolecules and metabolites. The specific interactions of nanoparticles with metabolites or biomacromolecules help, for example, simplify metabolomics spectra, improve the ionization efficiency for mass spectrometry or reveal relationships between spectral signals that belong to the same molecule. Lessons learned from nanoparticle-assisted metabolomics may also benefit other emerging areas, such as nanotoxicity and nanopharmaceutics.

## 1. Introduction

Metabolomics aims at the comprehensive characterization of all metabolites, i.e., molecules with a molecular weight <1500 Da, in a living system, such as a biofluid, cell lysate, tissue extract or entire organism [1,2,3]. Since metabolites are the products of a wide array of biochemical processes, information gained from metabolomics effectively complements other areas of systems biology, such as genomics, transcriptomics and proteomics [4]. Enabled by powerful modern analytical methods, in particular nuclear magnetic resonance (NMR) spectroscopy and mass spectrometry (MS), metabolomics studies can characterize phenotypes for a variety of different purposes, such as disease diagnosis and monitoring of treatment, ageing, the effect of food on health, toxicity studies via the exposome, or functional genomics [5,6,7]. Analysis of the metabolome is a complex task for a number of reasons. First, the estimated chemical composition by weight of living mammalian cells is approximately ~70% water and ~23% biomacromolecules and lipids, whereas less than ~7% belongs to the metabolome comprising many hundreds or thousands of different metabolites [8]. Second, metabolites display a diverse range of physical-chemical properties, such as molecular weight, chemical structure, stability, hydrophobicity, polarity and ionization efficiency [9]. These properties may play a role in whether a particular metabolite can be detected and quantified by a particular analytical technique. Because some metabolites can strongly bind to macromolecules, it can limit their detectability [10]. 

Ideally, a large number of metabolites is directly measured in a single biological sample requiring few, relatively simple sample preparation steps involving minimal physical separation. As the number of metabolomics studies is rapidly growing, current protocols manifest bottlenecks that need to be addressed. For sample preparation, they include the removal of background signals stemming from proteins and other biomacromolecules and the enhancement or suppression of signals that belong to metabolite targets. For data acquisition and analysis, the simplification of crowded spectra, the uniform and efficient ionization of metabolites and identification of different signals stemming from the same molecule are highly desirable. 

Advances in nanotechnology provide a wealth of opportunities for their application to biological systems, including metabolomics. The main characteristic of nanoparticles is that they have all three spatial dimensions confined to the 1–100-nm range. They can be categorized based on their atomic compositions (inorganic, organic and polycomponental), their shapes (nanospheres, nanocubes, nanorods and nanoshells) and their physical-chemical properties (optical, magnetic and electronic). Because of their chemical properties, large surface-to-volume ratio and unique quantum effects, nanoparticles often possess properties that qualitatively differ from their macroscopic counterparts, which makes them valuable for numerous applications, including ones used on an industrial scale. Over recent years, the application of nanotechnology to biological systems led to the emergence of new subfields, such as nanobiotechnology [11] and nanomedicine [12], which focus among others on the development of new bioanalytical methods, new therapeutics and new bioimaging agents. 

In order to continue the successful use of nanotechnology in biology, a better mechanistic understanding of the interactions between nanoparticles and biomolecules is clearly needed [13,14]. The advent of systems biology provides excellent opportunities to investigate biomolecule-nanoparticle interactions in different -omics fields. For example, studies of engineered nanoparticles that are able to differentially detect and alter the genome and proteome has been reported [15,16]. By contrast, metabolite-nanoparticle interactions have been much less explored. Because the metabolome directly reflects certain phenotypic traits and metabolites are often the initial, intermediate and end products of biological processes, it is expected that the better characterization of metabolite-nanoparticle interactions at atomic or near-atomic resolution will deepen our understanding of the effect and potential use of nanoparticles for a multitude of such processes. This knowledge may be key for the development of next-generation nanodevices that have minimal nanotoxicity. At the same time, the confluence of metabolomics and nanotechnology provides timely opportunities for the development of novel nanoparticle-based assays to facilitate the quantitative analysis of the metabolome of different types of biological samples. 

As the interest in metabolite-nanomaterial interaction continues to grow, the goal of this mini-review is to describe the present state and future prospects of the field. In contrast to DNA, metabolites display a large structural and chemical diversity, which presents unique challenges for their characterization. This makes certain approaches that are successfully used in other fields, such as the systematic labeling with fluorescent tags, impractical. For example, the quantitative binding affinities of the 20 natural amino acids to silica nanoparticle (SNPs) surfaces were recently determined based on ^13^C-NMR spin relaxation experiments [17]. The binding affinities across the different amino acids display a wide dynamic range, which illuminates how various physical-chemical factors dictate their binding properties. In this review, we will primarily focus on nano-assisted NMR and MS, since they are the two dominant platforms in metabolomics. They both yield rich spectra with detailed molecular information when directly applied to complex mixtures requiring little or no prior physical separation, and they are both suitable for high-throughput applications. Other analytical methods, including optical spectroscopy, colorimetric assays, electrochemical analysis or immunosensors, can provide valuable information that is complementary to NMR and MS [18,19]. A brief discussion of these methods is included in the following section.

## 2. Nanoparticles in Analytical Chemistry

Targeted metabolomics focuses on metabolites with a known molecular structure that have their spectra stored in readily accessible databases. The analysis of selectively chosen metabolites can be accomplished by the use of chemosensors. They have been well-established in analytical chemistry for the detection of specific target molecules, and their application to small molecules has been reviewed previously [18,19]. Similar strategies can be deployed for nanoparticles. For example, an important class of engineered nucleic acids or peptides that bind their target molecules with very high affinity and selectivity are aptamers. The specific targets of such sensors can be metabolites, such as adenosine triphosphate, theophylline, cocaine, histidine, argininamide, tyrosinamide, guanosine triphosphate or arginine [20]. In a recent study, a colorimetric aptasensor using unmodified gold nanoparticles (AuNPs) was reported for the simultaneous detection of multiple targets. Single-stranded DNA oligonucleotides were used as aptamer sensors for sulfadimethoxine, kanamycin and adenosine to stabilize AuNPs that were monodispersed in the absence of the target molecules. Upon sensing the presence of the small molecule targets, the aptamers preferentially bind to their targets, leaving the AuNPs unprotected, resulting in nanoparticle aggregation. Because the extent of AuNP aggregation is macroscopically manifested by distinct colors amenable to visual inspection, this strategy allows the sensitive and instrument-free detection of specific metabolites [21].

Surface enhanced Raman spectroscopy (SERS), which is an optical technique for molecular characterization based on the inelastic scattering of light by the analytes, allows the sensitive detection of molecules in the low nM range owing to the signal enhancement induced by a localized electromagnetic field around the plasmonic substrate. Due to their plasmonic surfaces, metallic nanoparticles are well-suitable materials and predominantly employed in SERS-based bioanalysis of a wide array of molecules. Recently, 3-mercaptophenylboronic acid (3-MPBA) modified AuNPs were reported to function as selective hydrogen peroxide (H_2_O_2_) probes for quantitative SERS detection both in vitro and in vivo. In the presence of H_2_O_2_, 3-MPBA is oxidized to 3-hydroxythiophenol, a change that can be sensitively monitored in SERS spectra with a detection limit of 70 nM. Moreover, when coupled with glucose oxidase that specifically reacts with glucose and yields H_2_O_2_, these substrates prove effective for the direct detection of glucose in complex metabolite mixtures, which was demonstrated for artificial urine and normal human serum samples [22]. In addition to primary metallic nanoparticles, core-shell nanoparticles are also gaining popularity to serve as enhancing substrates due to their multifunctionality, versatility and biocompatibility [23]. For example, AuNPs coated with SiO_2_ were used as a SERS substrate to monitor the composition of multiple purine compounds in bacterial metabolomes, which shows the potential of SERS as a technique for targeted analysis of certain metabolites with high sensitivity [24]. Importantly, only the analyte molecules that are bound or in close spatial vicinity to the plasmonic surface have their signal enhanced, whereas the effects from unadsorbed molecules are largely suppressed. This provides important opportunities to design and synthesize selective plasmonic nanostructures as SERS substrates for targeted metabolite analysis. Another nanoparticle-based method for immunoassays and sensors is resonance Rayleigh scattering applied to AuNPs as quenchers/enhancers in fluoroimmunoassays to monitor conjugated antigen-nanoparticle complexes for the identification of antibody biomarkers [25].

High-throughput screening of low molecular weight ligands of proteins is an important step during drug discovery. Interactions between proteins and certain metabolites can be studied with the use of nanoparticles. Some ligands can stabilize a protein in solution that otherwise has a strong propensity for binding to AuNPs, whereby both the bound and free state of the protein is characterized using the UV-Vis spectrum of the AuNPs [26]. Based on this approach, two transcription factors (FoxA1 and AP-2γ) together with human serum albumin (HSA) were screened for their binding properties to 14 small molecules and three known ligands for HSA. In these types of applications, the available large surface area for the attachment of antibodies facilitates analyte access to the antibodies. In addition, the nanoparticles provide signal amplification and label-free real-time protein detection. Nanoparticles thereby serve as probes of various macromolecule-metabolite interactions via their specific response to target small molecules. 

In addition to assisting the detection of analyte signals, nanoparticles can be used to improve sample preparation. Tailored functional nanoparticles were found to be highly effective in continuously harvesting, concentrating and preserving analytes present in the skin transudate for proteomics analysis [27]. The nanoparticles were made of a hydrogel with a molecular sieve as the shell and amino-containing dyes as high affinity baits in the core. The nanoparticles can: (1) target selected classes of proteins and peptides with specific dye chemistry immobilized in the particle core, providing very high affinity for analyte capture and preservation; (2) enrich the concentration of small proteins and peptides, while excluding large unwanted proteins in signal amplification; and (3) expand the detection limit of certain MS-based biomarker discovery platforms that use sequence analysis, multiple reaction monitoring or immunoassays. For example, this design is being used for the development of a skin patch to continuously harvest, concentrate and preserve proteins present in the skin transudate for proteomics analysis. With the continuous increase of known metabolite biomarkers, this type of point-of-care diagnostics may also take advantage of advances in nanoparticle-based metabolite detection. 

## 3. Nanoparticles for Metabolomics Sample Preparation 

Optimized sample preparation protocols are critical to remove negative interference with other signals in the spectrum stemming from molecules that belong to the complex biological matrix by either removing the macromolecules or by enriching the low concentration metabolites. Their well-controllable morphology, large reactive surface area and large variety of interactions make nanoparticles a promising tool for metabolite sample preparation. Until recently, nanoparticles have had little use in sample preparation for metabolomics. However, this situation is starting to change, as demonstrated by powerful synergies between these fields. Approaches described in this section were applied either to NMR or MS platforms. 

### 3.1. Silica Nanoparticles for Protein Removal and Metabolite Extraction in Protein-Rich Biofluids 

**Figure 1:**
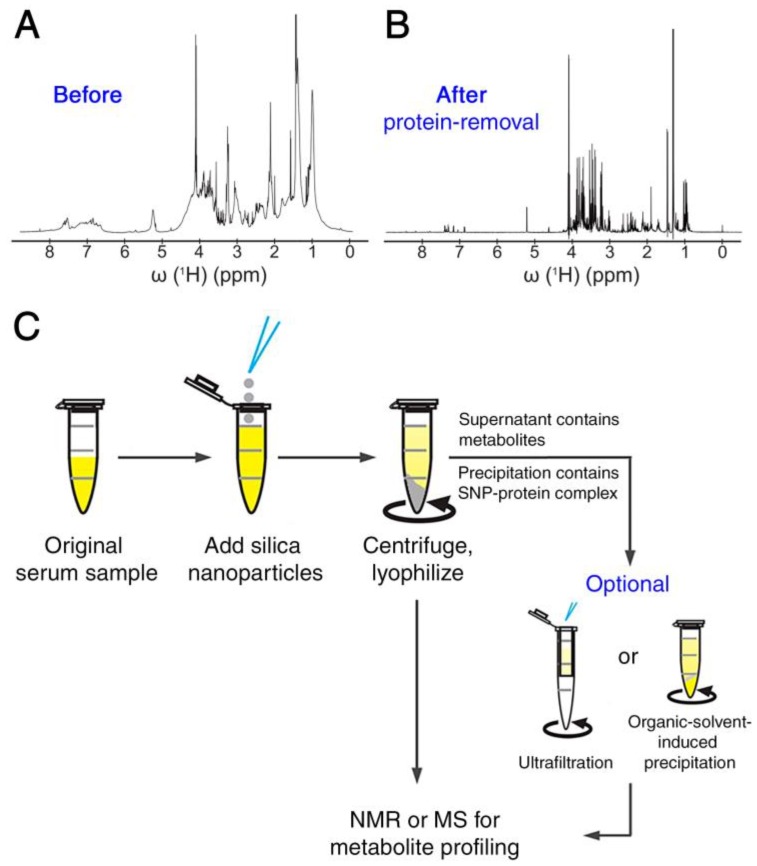
Protein removal from serum samples using silica nanoparticles for metabolomics studies. ^1^H-NMR spectra of metabolites in pooled human serum (**A**) before and (**B**) after removing proteins using silica nanoparticles. (**C**) The general workflow is depicted, which is efficient, cost effective and environmentally friendly and compares favorably with other protein removal methods. Reproduced with permission from [28].

Metabolite mixtures are usually extracted from biological matrices that also contain macromolecules, such as DNA, RNA, proteins and lipid bilayers. For example, it has been known that the large protein content of serum hampers the unambiguous detection of serum metabolites that show up in the NMR spectrum as sharp lines superimposed on the broad protein signal background resembling a distorted baseline (Figure 1A). Therefore, protein removal is a standard step prior to NMR data collection. Established methods for protein removal include organic solvent induced protein precipitation and ultrafiltration, which both face practical challenges (organic solvent removal, clogging of filters, etc.) [28,29]. We recently introduced an SNP-based method, which utilizes the electrostatic and hydrophobic interactions between surface amino acid residues and SNPs causing aggregation and co-precipitation of proteins and nanoparticles [28]. The technique permits the physical removal of the bulk of proteins, thereby significantly improving the quality of the metabolite NMR spectrum (Figure 1B). It can also be used as a quick pre-processing step in conjunction with the other protein removal methods, thereby increasing the protein removal efficiency (Figure 1C). The samples purified from proteins in this manner are amenable to quantitative NMR analysis. Considering that both solvent-induced precipitation and ultrafiltration are routinely used in MS-based analysis, nanoparticle (pre-)treatment should be equally suitable in this context. 

### 3.2. Nanoparticles for Solid-Phase Extraction for the Enrichment of Low Abundant Metabolites 

**Figure 2:**
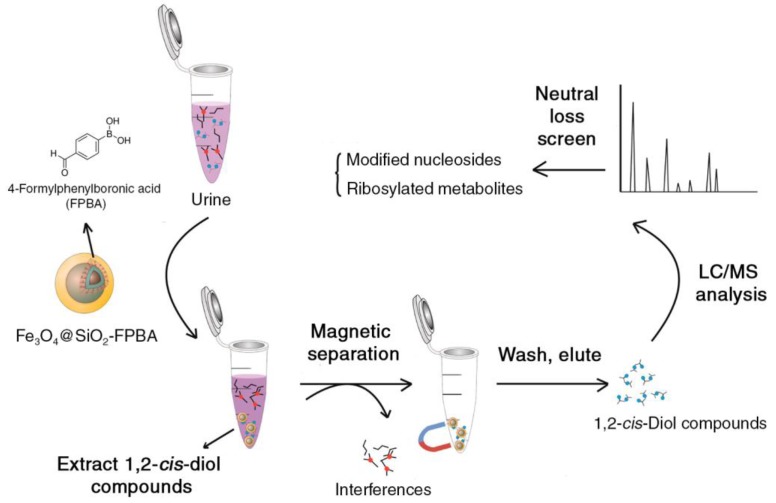
Enrichment of 1,2-*cis*-diol-containtng molecules from urine sample taking advantage of engineered magnetic nanoparticles for liquid chromatography (LC)-MS analysis. Figure adapted from [32] (Copyright © 2013, American Chemistry Society). FPBA, 4-formlyphenylboronic acid.

Solid-phase extraction (SPE) with nanoparticles is a rapidly growing subfield, which has been reviewed recently [30]. In the context of metabolomics, it is an emerging approach to selectively separate and enrich metabolites in complex mixtures. The underlying principle of SPE is that the adsorption of targeted analytes on a solid surface (stationary phase) from a solution (mobile phase) can be reversed through a second mobile phase based on physical-chemical properties of the metabolites adsorbed to the solid surface and the mobile phase. Compared to organic solvent extraction, selective metabolite extraction decreases overall metabolite coverage, but improves data quality through improved repeatability and reduced matrix effects [31]. For example, magnetic nanoparticles (MNPs) can be used as SPE sorbent, and they can also be surface modified to isolate and concentrate certain trace analytes of interest in complex matrices. In lieu of centrifugation or filtration, an external magnetic field is employed to separate the sorbent-analyte complexes from the solvent before the analytes are released and concentrated in a different solvent (Figure 2). Applications of MNPs initially focused on ribonucleic acids, deoxyribonucleic acids, proteins and peptides from viruses, bacteria and various biological fluids and subsequently also on small molecules. Ribosylated metabolites, including modified nucleosides, have also been tested for use as biomarkers for lymphoma, leukemia and breast cancer. The traditional extraction of ribosylated metabolites was accomplished through boronate materials, which have known limitations in terms of adsorption capacity and selectivity. An MNP was developed (Fe_3_O_4_@SiO_2_-FPBA) with 4-formlyphenylboronic acid (FPBA) grafted on its surface displaying a high density of boronate binding sites. This construct increased the adsorption capacity by 6–7-fold compared to the bulk reference material (Figure 2) [32].

A related approach is matrix solid-phase dispersion. For example, titanium dioxide nanoparticles (TNPs) were used as sorbent to bind phospholipids from olive fruit and oil in a lipidomics study [33]. Phospholipids are nutrients involved in lipid digestion, absorption, transport and many other signaling pathways. Identification and characterization of phospholipids in Mediterranean food is of interest because of its many potential health benefits. However, this process is impeded by the existence of a high abundance of glycerides in these samples and a high variety of fatty-acyl chains in each phospholipid species. ^31^P-NMR spectroscopy was found to be suitable for the detection and quantification of phospholipids in olive oils without separation [34]. In order to analyze phospholipids by matrix-assisted laser desorption/ionization time-of-fly (MALDI-TOF) MS, TNPs were found to be an efficient dispersion sorbent, which is able to selectively interact with the phosphate group of phospholipids via a chelating bidentate bond. To separate phospholipids from these food matrices, the samples were mixed with TNPs and powderized, packed into a column, washed with weakly acidified water, followed by elution with a chloroform/methanol mixture. This procedure reduced the extraction time and organic solvent usage in comparison with the traditional Bligh and Dyer extraction method where high levels of glycerides are produced and signals of phospholipids are suppressed.

## 4. Nanoparticle-Metabolite Interaction Assist Metabolite Detection

The sensitivity of NMR spectra is directly related to the transverse spin relaxation properties of the detected nuclei. For molecules in solution, spin relaxation depends on the rotational tumbling rate, which is a function of the molecular size, the solvent viscosity and the transient interactions between the molecules with additives, such as nanoparticles, via electrostatic and hydrophobic interactions. Therefore, the addition of a colloidal nanoparticle suspension to solution NMR samples can cause the attenuation of the NMR signals of those metabolites that selectively interact with the nanoparticles. MS, on the other hand, requires both desorption and ionization of the analytes. The successful formation of ions using electrospray ionization depends on two steps: the transfer of the compound of interest into the gas phase and the creation of a charge (in case it does not already possess a net charge) [35]. Nanoparticles that were modified to have specific electric or thermal properties can facilitate the ionization of certain small molecules in their vicinity. Recent applications of nanoparticle-assisted NMR and MS detection are discussed in the following. 

### 4.1. Silica Nanoparticles for NMR Spectra Simplification

**Figure 3:**
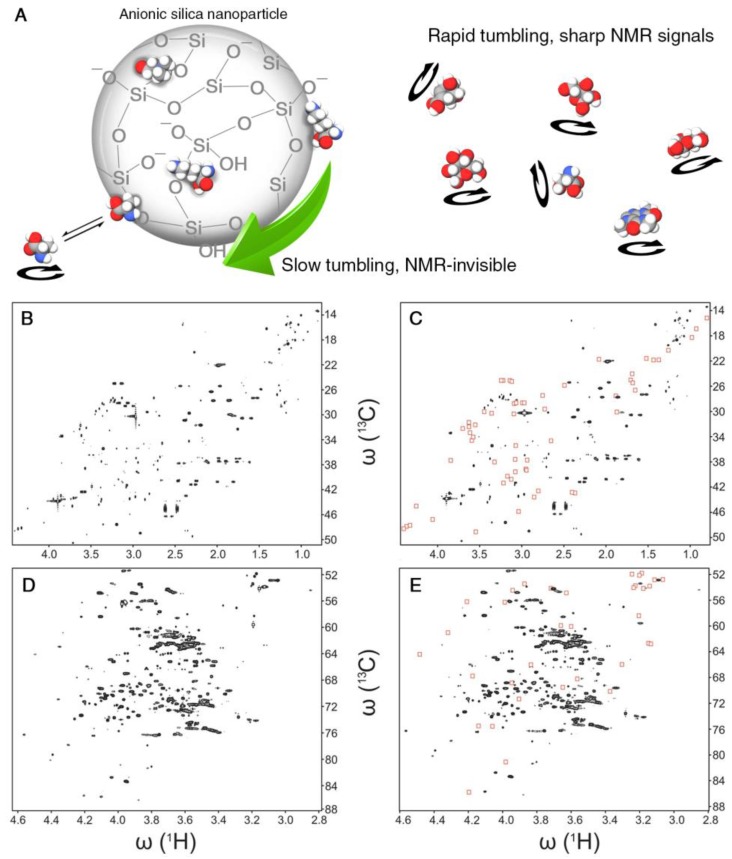
(**A**) Schematic representation of the effect of nanoparticle-metabolite interactions on differential line broadening in NMR spectra. Anionic silica nanoparticles adsorb positively-charged metabolites, slow down their tumbling rates and hence broaden and diminish the corresponding spectral peaks. Even transiently-interacting metabolites are subject to potentially strong line-broadening effects. The linewidths of non-interacting metabolites, however, are not affected. (**B**–**E**) Two representative 2D ^13^C-^1^H heteronuclear single quantum correlation (HSQC) NMR spectral regions of pooled human urine sample in the absence and presence of nanoparticles. A subset of cross-peaks (highlighted by red boxes in (**C**,**E**)) that primarily belong to positively charged metabolites is suppressed by the presence of anionic silica nanoparticles compared to the nanoparticle-free sample (**B**,**D**). Adapted with permission from [36].

Many metabolomics samples display peak overlaps in their NMR spectra posing a challenge for the comprehensive and accurate identification of the underlying metabolites. This can be a severe obstacle for the interpretation of 1D NMR spectra and sometimes also 2D spectra. The large surface-to-volume ratio of nanoparticles enables efficient interactions with biological molecules even in the presence of modest amounts of nanoparticles. Certain nanoparticles can be uniformly dispersed as colloids that are stable for weeks without aggregation or precipitation. Different surface modifications of nanoparticles can be used to selectively probe the presence of biological molecules based on their physical-chemical properties. In an NMR-based metabolomics study, two types of SNPs were employed. The pristine surface of anionic SNPs contains siloxane and silanol (Si-OH) groups. At physiological pH, the latter are partially dissociated, giving rise to a negative net surface charge and, hence, negative ζ-potential [36]. The surface of cationic SNPs, on the other hand, is doped with a monolayer of alumina that is positively charged. For SNPs with a diameter of 20 nm, the rotational tumbling is approximately 1 μs according to the Stokes–Einstein–Debye relationship. When adding either anionic or cationic SNPs to a metabolite mixture, metabolites with an opposite charge experience an attractive interaction leading to a bound or transiently bound state to the surface of the nanoparticle, which causes a dramatic slow-down of their rotational tumbling rates and NMR-line broadening accompanied by the weakening or complete suppression of the NMR signals of the bound metabolites (Figure 3A). Comparison of the spectrum collected in the presence of SNPs with the original spectrum directly highlights NMR resonances of charged metabolites, thereby assisting their identification and physical-chemical characterization in terms of electric charges (Figure 3B–E). Moreover, certain nanoparticles have hydrophobic surface patches that preferentially interact with metabolites that possess hydrophobic groups [37], such as methyl group clusters, providing additional information about the physical-chemical properties of metabolites even when they are “unknowns”, i.e., their structure is not known and their spectral information has not (yet) been compiled in metabolomics databases. Physical separation of metabolomics samples by chromatographic techniques is rather time consuming, as it requires the measurement of at least one 2D NMR spectrum for each fraction. By contrast, the addition of small amounts of SNPs to the NMR sample converts the NMR tube into a “transient” affinity column, thereby altering the NMR spectrum to facilitate its analysis. At constant pH, NMR is inherently insensitive to electrostatic charges, whereas the addition of SNPs manifests molecular charges through the presence and disappearance of NMR signals. Future progress can entail the synthesis of specifically functionalized nanoparticles so that they differentially interact with metabolites that have the same carbon backbone, but different functional groups, such as phosphate groups, aldehydes, alcohols, methyl groups, etc. Nanotoxicity studies found that the surface charge of nanoparticles can have a critical impact on cellular toxicity [38]. Therefore, a thorough understanding of the driving forces of the interactions of nanoparticles with other molecules will provide a better understanding of the underlying chemical mechanisms of toxicity. 

### 4.2. Nanoparticles to Establish Intra-Molecular Correlations between NMR Signals

Translational molecular diffusion can be sensitively monitored by NMR using pulsed-field gradients [39]. Because the translational diffusion coefficient in solution depends on the size and shape of a molecule, it can be used to separate those NMR signals of a complex mixture that belong to different molecules by diffusion-ordered spectroscopy (DOSY) [40]. The method requires that different molecules have a sufficiently distinct diffusion coefficient, which is not always the case. Therefore, a matrix is sometimes introduced to enhance the separation, which is known as “matrix-assisted DOSY” [41]. Due to their relatively large size, nanoparticles are slowly-diffusing species and dependent on the strengths of their interactions with certain metabolites, and they can differentially slow down the metabolites’ average translational diffusion, which can be used as a “diffusion filter” to separate metabolite signals. Recently, AuNPs functionalized with Zn^2+^-triazacyclonane complexes were applied to detect phosphorylated organic molecules to measure, for example, betamethasone phosphate in commercial drug tablets [42]. AuNPs with a 2-nm diameter were coated with thiols with different functional groups, which non-covalently interact with small organic molecules and slow down their translational diffusion. The residual reorientational mobility of the organic analytes produce NMR spectra with relatively narrow linewidths allowing the quantitative measurement of the NMR amplitude reduction caused by the molecular interactions, which are driven by (1) the ion pairing/coordination interactions between negatively-charged phosphate esters and the metal ion and (2) the hydrophobic interactions between the inner monolayer and the organic moieties attached to the phosphate group. In a mixture with four compounds (diphenyl phosphate, benzoate, arbutin and tyramine) with similar translational diffusion coefficients (5 − 7 × 10^−10^ m^2^ s^−1^) in aqueous solution, the addition of AuNPs significantly decreased the diffusion coefficients of benzoate (3.6 × 10^−10^ m^2^ s^−1^) and diphenyl phosphate (1.9 × 10^−10^ m^2^ s^−1^), but not the others. 

**Figure 4:**
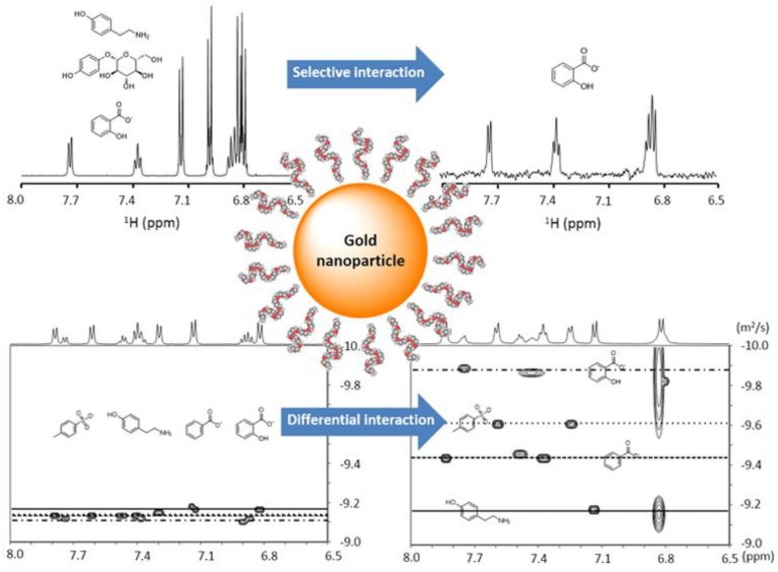
Application of nanoparticles with different surface modifications for selective molecular sensing based on NMR magnetization transfer or diffusion-ordered spectroscopy (DOSY). The interaction strength and specificity between the surface coating ligands and organic molecule analytes can be modulated via rationally-designed nanoparticle surface modifications, reflecting the potential of this method for the selective detection and identification of metabolites in a complex biological mixture. Reprinted with permission from [43].

The presence of interactions between metabolites and macromolecules can be enhanced by coating nanoparticles with the macromolecules and using a nuclear Overhauser effect (NOE) pumping experiment to detect those metabolites in a mixture that interact with the macromolecules in fast exchange [43,44]. Because a non-covalent analyte receptor is introduced, this method was termed “NMR chemosensing” as a protocol for analyte identification and quantification in complex mixtures. The method relies on two different NMR experiments. First, when the interaction is selective and the exchange is fast on the diffusion timescale, the nuclear spins located on the NP monolayer can be used as a magnetization source to be transferred to the analytes via NOE pumping highlighting only those metabolites that transiently interact with the nanoparticle surface. Second, when the interactions are sufficiently strong, a slow-down of the translational diffusion of the analyte can be observed, allowing for optimal separation of the different analytes in a DOSY experiment (Figure 4).

In NMR-based metabolomics, an important task is the unambiguous identification of NMR peaks that belong to the same molecule. This is important for both the correct identification of metabolites by database query and for the characterization of unknown metabolites. For molecules with a single spin system, 2D total correlation spectroscopy (TOCSY) NMR experiments are very powerful as they produce cross-peaks between all the spins of the same spin system [45]. For molecules with multiple spin systems, TOCSY will only permit the identification of individual spin systems, but it will not provide information about whether two spin systems belong to the same molecule. DOSY-type experiments applied to complex mixtures, on the other hand, may not have sufficient resolution along the diffusion dimension to discriminate between different molecules. An approach to address this situation has been proposed recently, which is based on complementary ion exchange resin beads (e.g., 30–300 μm) to differentially attenuate 2D NMR cross-peaks that belong to different metabolites [46]. Based on their characteristic attenuation patterns, cross-peaks could be clustered and assigned to individual molecules, including metabolites with multiple spin systems, as demonstrated for a metabolite model mixture and *E. coli* cell lysate. This approach, which is independent of spin magnetization transfer across the molecules, can be applied to both 1D and 2D NMR spectra. It provides unique information for the determination of unknown metabolites. Although the study focused on anionic and cationic resin beads, it seems feasible to use nanoparticles incorporated into resin materials for the same purpose, and stronger differential attenuation may be realized through specifically functionalized nanoparticles that recognize groups of metabolites based on their unique physical-chemical properties [47,48]. 

### 4.3. Nano-Scaled Surface Structure for Ionization Enhancement in Mass Spectrometry

**Figure 5:**
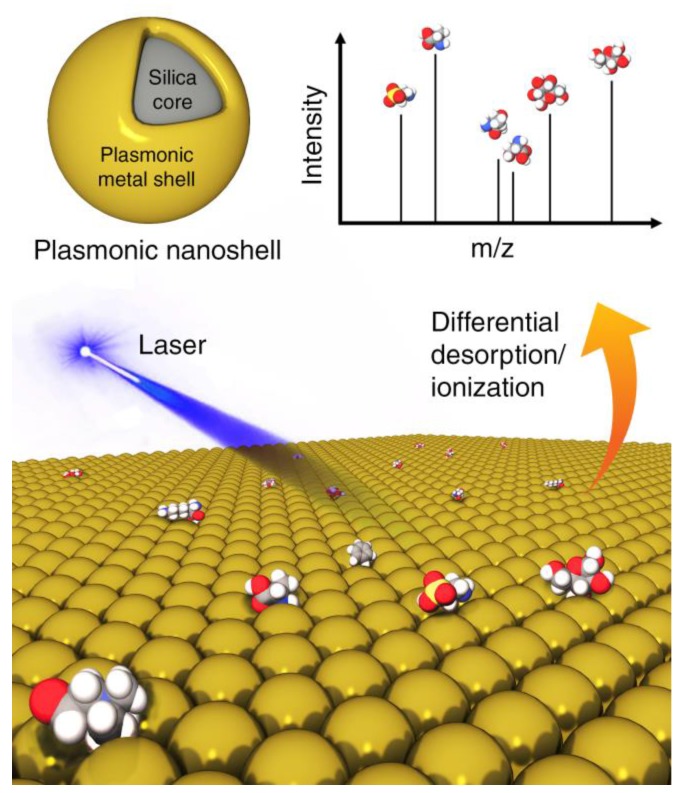
Inorganic nanoparticles, such as plasmonic nanoshells, enable enhanced ionization of small molecules in matrix-assisted laser desorption/ionization mass spectrometry (MALDI-MS) for metabolomics applications.

Analogous challenges exist for MS-based metabolomics applications. Certain metabolites, even when abundantly present, will not easily ionize or desorb. Efforts have been made through the use of nanoparticles with modified surfaces to overcome these issues. Conventional MALDI-MS uses a protonated matrix (e.g., organic solvent) to assist ionization of macromolecules (e.g., polymers and proteins) by forming micro-crystals to protect the analytes from the laser irradiation to absorb photon energy. Because for low-mass analytes, matrix ion interference and detector saturation are unavoidable, significant efforts have been devoted to eliminate matrix ion interference by means of different matrix substances. Other energy-absorbing materials have been proposed as matrix alternatives for laser desorption/ionization (LDI) techniques for MS measurements of metabolites including C_60_ fullerenes, SNPs and carbon nanotubes [49]. They provide a clean spectral background and a larger capacity to ionize metabolites. In fact, this method has also been referred to as surface-assisted laser desorption/ionization mass spectrometry (SALDI-MS) where the nanoparticles were highlighted (Figure 5) [50]. It has been found that material functionalities and morphologies, including particle size and porosity, all play important roles in their impact on the LDI process since the unique electrical, thermal and optical properties of these surfaces permit the efficient transfer of the laser energy to the analytes to generate strong MS signals. In a recent application, plasmonic nanoshells were reported to enhance LDI-MS detection of amino acids in serum [51] due to four reasons: (1) avoidance of strong interference in the low mass range (*m*/*z* < 500) and sweet spot effects by traditional organic matrices; (2) high absorption coefficient in the UV-Vis optical range; (3) cost-friendly and facile preparation for large-scale application; (4) mature surface modification protocol based on the gold-thiol (Au-S) interaction for capturing analytes. In particular, recent theoretical and experimental findings have demonstrated that plasmonic nanoparticles can directly convert the collected light into hot carriers with tunable plasmon resonances. Low detection limits of various amino acids (3–30 pmol) were achieved by gold nanoshells with superior properties compared to gold nanorods and nanospheres. A more focused discussion of inorganic nanomaterials for LDI-MS analysis of small molecules can be found recently [52]. 

MS-based imaging of tissues is another popular application. In MALDI, the formation of a homogeneous layer of matrix/analyte crystals that are smaller than the tissue area of interest is required. As an alternative, metal ions or nanoparticles were introduced for imaging in matrix free methods for increased spatial resolution and sensitivity. For example, nano-scale complexes of graphite, silver or magnetic nanoparticles with analytes were prepared for detection [50]. Nanoparticles can recognize cell organelles, proteins and other macromolecules, whereby the tissue and cell distribution of nanoparticles can be regulated to provide single cell spatial resolution. Nanoparticles in MALDI, LDI, as well as MS imaging have been found to provide advantages by increasing the ionization efficiency and handling of limited sample volumes and to provide improved spatial information. 

## 5. Conclusions and Future Perspective

Metabolomics is a fast-growing, highly cross-disciplinary field of research. Synergistic developments in biomedicine, biotechnology, analytical instrumentation and bioinformatics have all contributed to the advancement of this field. Materials science, especially nanotechnology, has only started to have an impact on metabolomics as discussed in this review. We described how nanoparticle-assisted technologies have the potential for future advances or even breakthroughs in NMR- and MS-based metabolomics. 

Although still at an early stage, nanoparticles with electric surface charges, hydrophobic surfaces or specific chemical reactivity can find useful applications for the measurement and analysis of metabolomics samples. During sample preparation, the extraction, separation, isolation and enrichment of analytes can be both challenging and critical for the analysis of complex matrices of environmental, food or biological samples by NMR and MS. Nanoparticles can be effectively applied to these steps requiring minimal manipulation of the sample. For data acquisition, nanoparticles can serve as efficient, low-cost and environmentally-friendly components that help control the composition of metabolic mixtures in solution. Nanoparticles can help simplify the spectrum and differentiate peaks based on spin relaxation or ionization properties, thereby producing more informative spectra that help uncover the structures and other properties of metabolites. Importantly, as nanotechnology continues to grow, novel nanomaterials are fabricated on an industrial scale for numerous applications. As a result, functional nanoparticles coupled with various surface modifications are becoming more accessible and affordable, which greatly benefits nanoparticle-assisted metabolomics and beyond. A predictive understanding of the complex interactions between metabolites and nanoparticles, however, will need to be systematically established.

As important challenges in metabolomics are being tackled, new capabilities are being uncovered, including structure determination of unknown metabolites with little or no mixture separation, absolute concentration measurements, and synergistic uses of NMR and MS, in combination also with other techniques. New applications of nanotechnology are likely to emerge that will drive the development of analytical methods with better sensitivity, resolution, quantification and throughput.
